# *CYP2E1* C-1054T and 96-bp I/D genetic variations and risk of gestational diabetes mellitus in chinese women: a case-control study

**DOI:** 10.1186/s12884-023-05742-y

**Published:** 2023-06-01

**Authors:** Yifu Pu, Qingqing Liu, Kaifeng Hu, Xinghui Liu, Huai Bai, Yujie Wu, Mi Zhou, Ping Fan

**Affiliations:** 1 Laboratory of Genetic Disease and Perinatal Medicine, Key Laboratory of Birth Defects and Related Diseases of Women and Children, Ministry of Education, West China Second University Hospital, Sichuan University, Chengdu, Sichuan 610041 People’s Republic of China; 2 Department of Obstetrics and Gynecology, West China Second University Hospital, Sichuan University, Chengdu, Sichuan 610041 People’s Republic of China

**Keywords:** Gestational diabetes mellitus, CYP2E1, Cytochrome P450, Genetic polymorphism, Xenobiotic metabolism, Oxidative stress

## Abstract

**Background:**

Cytochrome P450 2E1 (CYP2E1) plays a key role in the metabolism of xenobiotic and endogenous low-molecular-weight compounds. This study aimed to determine if the genetic variations of 96-bp insertion/deletion (I/D) and C-1054T (rs2031920) in *CYP2E1* were associated with the risk of gestational diabetes mellitus (GDM).

**Methods:**

*CYP2E1* polymorphisms were genotyped in a case-control study of 1,134 women with uncomplicated pregnancies and 723 women with GDM. The effects of genotype on the clinical, metabolic, and oxidative stress indices were assessed.

**Results:**

The *CYP2E1* C-1054T variant was associated with an increased risk of GDM based on the genotype, recessive, dominant, and allele genetic models (*P* < 0.05). The TT + CT genotype remained a significant predictive factor for GDM risk after correcting for maternal age and pre-pregnancy body mass index (OR = 1.277, 95% CI: 1.042–1.563, *P* = 0.018). Moreover, fasting insulin concentrations and homeostatic model assessment of insulin resistance were significantly higher in GDM patients carrying the T allele than in those with the CC genotype (*P* < 0.05). Furthermore, the combined genotype II + ID/TT + CT of the 96-bp I/D and C-1054T polymorphisms further increased the risk of GDM when the combined genotype DD/CC was set as the reference category (OR = 1.676, 95% CI: 1.182–2.376, *P* = 0.004).

**Conclusions:**

The T allele of the C-1054T polymorphism and its combination with the I allele of the 96-bp I/D variation in *CYP2E1* are associated with an increased risk of GDM in the Chinese population. The − 1054T allele may be associated with more serious insulin resistance in patients.

## Background

Gestational diabetes mellitus (GDM) is one of the most common gestational complications. It is characterized by carbohydrate intolerance leading to hyperglycemia with an onset or first identification during pregnancy [1, 2]. It is a growing health concern in pregnancy because it impairs the health of several million women worldwide [1, 3]. The incidence of GDM varies from 5 to 25.5% globally depending on the diagnostic criteria, ethnic group, age, and body mass index (BMI) [2, 4, 5]. Its prevalence in China is 14.8% [4]. GDM may result in unfavorable pregnancy outcomes in both the mother and infant, including macrosomia, neonatal hypoglycemia, higher cesarean rate, and preeclampsia [2, 6, 7]. It is associated with increased long-term health risks, including type 2 diabetes mellitus and cardiovascular diseases in mothers, and metabolic syndrome, overweight, and obesity in both the mother and offspring [3, 7–10]. The etiology of GDM is unknown and may be related to genetic variants [11–13], increased oxidative stress [11, 14–16], dyslipidemia [17], chronic inflammation [18], abnormal expression of placental hormones and cytokines [19–21], and assisted reproduction technology [22].

Cytochrome P450 2E1 (CYP2E1) belongs to the cytochrome P450 family [23]. It is an abundant enzyme that accounts for approximately 21% of all CYP proteins in the human liver [24]. It can metabolize various low-molecular-weight xenobiotics, including medications and environmental toxins, and endogenous compounds to their highly active intermediate metabolites during phase I metabolic reactions [23]. These high-reactivity intermediates are then combined with hydrophilic molecules or chemical groups during phase II metabolic reactions and converted into water-soluble and non-toxic metabolites [23]. Nevertheless, some active intermediates, including reactive oxygen species and carcinogenic or hepatotoxic metabolites, can covalently conjugate with biological macromolecules, influence the function and molecular framework of these biomolecules, and play key roles in the development of some cancers, alcohol or drug-induced liver impairment, and non-alcoholic fatty liver disease [23, 25, 26]. Moreover, CYP2E1 has been reported to participate in the metabolism of some fatty acids such as arachidonic acid, which may affect signal transduction and cellular homeostasis [23].

CYP2E1 is a 493-amino acid protein encoded by *CYP2E1* [27]. Genetic polymorphisms, such as the 96-bp insertion/deletion (I/D) and C-1054T (RsaI, rs2031920) in the 5′-flanking regulatory region of *CYP2E1* may affect the transcriptional activity of CYP2E1 [28–30]. Usually, the CYP2E1*5A or RsaI wild-type (c1) allele refers to the C allele of the single nucleotide polymorphism (SNP) C-1054T, whereas the CYP2E1*5B or RsaI variant c2 allele represents the T allele [23, 31]. There is almost a complete link disequilibrium between the 96-bp I/D and C-1054T variations (D′ = 0.94) [32]. Notably, these two polymorphisms are associated with the occurrence of some cancers [31–33], adverse birth outcomes [34], polycystic ovary syndrome (PCOS) [27], and drug-induced liver injury [35].

CYP2E1 catalyzes the production of reactive intermediates from xenobiotics and endogenous substances. These intermediates may damage the structure and function of biomacromolecules, resulting in increased oxidative stress, epigenetic changes, cell dysfunction, and apoptosis of cells [23, 26, 36]. Thus, CYP2E1 may be involved in the pathogenesis of GDM. However, limited data are available on the relationship between CYP2E1 and GDM, and it remains unknown whether the C-1054T and 96-bp I/D genetic variations in *CYP2E1* are associated with GDM. The present study explored the association between these two genetic polymorphisms and the risk of GDM, and assessed the effect of genotype on oxidative stress and clinical and metabolic parameters in the Chinese population.

## Methods

### Study subjects

This case-control study included 723 patients with GDM and 1,134 controls. All participants were recruited from the Department of Obstetrics and Gynecology of the West China Second University Hospital between 2013 and 2021. This study was conducted in accordance with the principles of the Declaration of Helsinki. Written informed consent was obtained from all the study subjects. The study was approved by the Institutional Review Board of West China Second University Hospital, Sichuan University (approval numbers: 2020-036 to Ping Fan and 2017- 033 to Xinghui Liu).

At 24–28 gestational weeks, each pregnant woman underwent a routine 75 g oral glucose tolerance test. GDM was diagnosed based on the guidelines of the International Association of Diabetes Pregnancy Study Groups by a woman having one or more of the following findings: fasting glucose ≥ 5.1 mmol/L; 1 h glucose ≥ 10.0 mmol/L; or 2 h glucose ≥ 8.5 mmol/L [37]. Control participants with uncomplicated pregnancies were enrolled at the same hospital during the same period. The inclusion criterion for participants was singleton pregnancy.

The exclusion criteria were chronic hypertension; diabetes mellitus before pregnancy; twin/multiple pregnancies; preeclampsia; intrahepatic cholestasis of pregnancy; and autoimmune, renal, cardiac, hepatic, and other endocrine disorders. Women who had premature deliveries or underwent in vitro fertilization were excluded from the control group.

Clinical and anthropometric variables of the participants, including systolic blood pressure (SBP), diastolic blood pressure (DBP), BMI (kg/m^2^), gestational age, and birth height and weight of infants were measured or assessed.

Blood samples were obtained after at least 8 h of fasting during the third trimester of pregnancy or before delivery, kept on ice, and centrifuged at 1500 × *g* for 15 min at 4 °C within 2 h. Plasma and serum aliquots were stored at -80 °C for later analysis. Blood cells in EDTA anticoagulant tubes were stored at 4 °C before deoxyribonucleic acid (DNA) extraction.

### Analysis of metabolic and oxidative stress parameters

Serum total cholesterol (TC), high-density lipoprotein cholesterol (HDL-C), low-density lipoprotein cholesterol (LDL-C), triglyceride (TG), apolipoprotein (apo)A1, apoB, plasma insulin and glucose concentrations, malondialdehyde (MDA), total oxidant status (TOS), total antioxidant capacity (TAC), oxidative stress index (OSI; i.e., TOS/TAC ratio), and homeostatic model assessment of insulin resistance (HOMA-IR) were measured or evaluated as previously described [14, 38, 39]. The intra- and inter-assay coefficients of variation for all measurements did not exceed 5% and 10%, respectively.

### DNA extraction and genotyping

Genomic DNA was extracted from the leukocytes of participants using a routine method. CYP2E1 genetic polymorphisms were genotyped using polymerase chain reaction (PCR) and/or restriction fragment length polymorphism methods as previously described [27]. To guarantee genotyping quality, another operator randomly re-genotyped approximately 30% of the DNA samples and the results of the two genotypes were identical.

### Statistical analyses

All statistical analyses were conducted using Statistical Program for Social Sciences (SPSS) version 21.0 (IBM SPSS Statistics, IBM Corporation, Armonk, New York, USA). Data are expressed as the mean ± standard deviation. Hardy-Weinberg equilibrium was tested in cases and controls using chi-square (χ^2^) analysis. Allelic and genotypic frequencies in different genetic models were compared between the cases and controls using the χ^2^ test. The differences in variables between GDM and control were estimated using an independent-sample Student’s *t*-test or a non-parametric test (for variables with an asymmetric distribution). Analysis of covariance was used to assess differences in biochemical parameters between the groups after correcting for differences in age and pre-pregnancy BMI. Odds ratios (OR) and 95% confidence intervals (CI) were used to evaluate the risk of GDM associated with *CYP2E1* genetic variants using a logistic regression method or the χ^2^ test. The effect of genotype, GDM status and their interaction was evaluated by a two-way univariate general linear model. Statistical significance was set at *P*-value < 0.05.

The power value due to the minor allele frequency of *CYP2E1* C-1054T SNP and sample size was determined according to a previously described method [27]. The analysis of linkage disequilibrium between the 96-bp I/D and C-1054T variants was conducted by the SHEsis online software at http://analysis.bio-x.cn/myAnalysis.php.

## Results

### Clinical and biochemical properties of the participants

As shown in Table 1, the pre-pregnancy BMI was higher in the GDM group than in the control group. Among the 723 patients, 81 required insulin therapy, whereas the remaining patients only underwent lifestyle modifications. After correcting for differences in age and pre-pregnancy BMI, fasting Glu and Ins concentrations, HOMA-IR, TG, TG/HDL-C ratio, apoB/apoA1 ratio, MDA, TOS, and OSI were significantly higher, whereas LDL-C and apoA1 concentrations, weight gain during pregnancy, gestational age (days), and neonatal birth weight and height were significantly lower in the GDM group than in the control group.

**Table 1:** Clinical, metabolic, and oxidative stress parameters in patients with GDM and control women

	Controls (n = 1134)	GDM (n = 723)	*P*	*P* ^*a*^
**Clinical characteristics**				
Age (years)	35.53 ± 3.68	35.60 ± 4.03	0.701	
Pre-pregnancy BMI (kg/m^2^)	21.25 ± 2.68	22.27 ± 2.93	<0.001	
Delivery BMI (kg/m2)	26.73 ± 2.71	26.84 ± 3.18	0.449	
Weight gain during pregnancy (kg)	13.98 ± 4.26	11.50 ± 4.20	< 0.001	< 0.001
SBP (mmHg)	115.21 ± 10.15	115.69 ± 11.86	0.352	0.976
DBP (mmHg)	72.19 ± 8.00	72.72 ± 9.01	0.200	0.354
Gestational age (days)	274.77 ± 6.23	272.22 ± 12.61	< 0.001	< 0.001
Parity	1.62 ± 0.54	1.58 ± 0.53	0.188	0.008
Neonatal birth height (cm)	49.87 ± 1.92	49.61 ± 1.84	0.005	0.002
Neonatal birth weight (g)	3383.06 ± 376.11	3335.13 ± 442.36	0.016	0.001
Macrosomia % (n)	4.4 (50)	5.4 (39)	0.333	
Insulin treatment (n)	0	81		
Metabolic profile*				
Fasting Glu (mmol/L)	4.35 ± 0.43	4.62 ± 0.81	< 0.001	< 0.001
Fasting Ins (pmol/L)	72.58 ± 35.98	106.61 ± 149.53	< 0.001	< 0.001
HOMA-IR	2.05 ± 1.09	3.65 ± 9.09	< 0.001	< 0.001
TG (mmol/L)	3.63 ± 1.40	3.91 ± 1.68	< 0.001	0.007
TC (mmol/L)	6.06 ± 1.08	5.96 ± 1.10	0.064	0.322
HDL-C (mmol/L)	1.99 ± 0.41	1.97 ± 0.43	0.337	0.968
LDL-C (mmol/L)	3.17 ± 0.99	2.97 ± 0.97	< 0.001	0.001
TG/HDL-C	1.92 ± 0.88	2.10 ± 1.10	< 0.001	0.008
ApoA1 (g/L)	2.37 ± 0.44	2.30 ± 0.42	< 0.001	0.002
ApoB (g/L)	1.15 ± 0.26	1.15 ± 0.26	0.751	0.359
ApoB/apoA1	0.50 ± 0.15	0.51 ± 0.13	0.038	0.016
Oxidative stress indices**				
TOS (µmol H_2_O_2_ Equiv./L)	20.98 ± 6.97	25.91 ± 10.56	< 0.001	< 0.001
TAC (mmol Trolox Equiv./L)	1.11 ± 0.19	1.12 ± 0.21	0.190	0.835
OSI	19.36 ± 7.29	23.32 ± 10.11	< 0.001	< 0.001
MDA (nmol/ml)	5.37 ± 1.21	5.88 ± 1.43	< 0.001	< 0.001

Values are presented as mean ± SD. The frequency of macrosomia was compared by chi-squared tests

BMI, body mass index; SBP, systolic blood pressure; DBP, diastolic blood pressure; Glu, glucose; Ins, insulin; HOMA-IR, homeostatic model assessment of insulin resistance; TG, triglycerides; TC, total cholesterol; HDL-C, high-density lipoprotein cholesterol; LDL-C, low-density lipoprotein cholesterol; apo, apolipoprotein; TOS, total oxidant status; TAC, total antioxidant capacity; MDA, malondialdehyde; OSI, oxidative stress index

^*a*^ All comparisons of parameters were corrected for differences in age and pre-pregnancy BMI.

*Controls: n = 1071; GDM: n = 674

**Controls: n = 849; GDM: n = 557

### ***CYP2E1*96-bp I/D and C-1054T genotypic and allelic frequencies**

Genotypic frequencies of the 96-bp I/D and C-1054T variants were in accordance with Hardy–Weinberg equilibrium in both the GDM and control groups (all *P* > 0.05). There is a reasonably high linkage disequilibrium between the C-1054T and 96-bp I/D variants (D′ = 0.943). As shown in Table 2, the frequencies of the TT genotype (5.7 vs. 3.5%), CT genotype (32.8 vs. 29.3%), and T allele (22.1 vs. 18.2%) of the C-1054T SNP were significantly higher in patients with GDM than in the control group (OR = 1.644, 95% CI:1.053–2.568, *P* = 0.027 for the recessive model; OR = 1.280, 95% CI:1.054–1.554, *P* = 0.013 for the dominant model; OR = 1.275, 95% CI:1.082–1.502, *P* = 0.004 for the allele model). The TT + CT genotype had a significant predictive role for GDM risk after correcting for differences in age and pre-pregnancy BMI (OR = 1.277, 95% CI: 1.042–1.563, *P* = 0.018). The statistical power to discern an inheritance correlation was 0.939 for C-1054T variation (prevalence = 0.15; significance level = 0.05). No significant differences were identified between case and control subjects based on the different genetic models for the 96-bp I/D variation (*P* > 0.05, Table 2).

**Table 2:** Association of *CYP2E1* C-1054T and 96-bp I/D polymorphisms with GDM using different genetic models

	Controls (n = 1134)	GDM (n = 723)	*x* ^*2*^	*P*
**C-1054T**				
Genotype				
CC	762 (67.2%)	445 (61.5%)		
CT	332 (29.3%)	237 (32.8%)		
TT	40 (3.5%)	41 (5.7%)	8.585	0.014
Recessive				
CC + CT	1094 (96.5%)	682 (94.3%)		
TT	40 (3.5%)	41 (5.7%)	4.863	0.027*
Dominant				
CC	762 (67.2%)	445 (61.5%)		
TT + CT	372 (32.8%)	278 (38.5%)	6.188	0.013**
Allele				
C	1856 (81.8%)	1127 (77.9%)		
T	412 (18.2%)	319 (22.1%)	8.474	0.004***
**96-bpI/D**				
Genotype				
DD	710 (62.6%)	440 (60.9%)		
ID	372 (32.8%)	253 (35.0%)		
II	52 (4.6%)	30 (4.1%)	1.038	0.595
Recessive				
DD + ID	1082 (95.4%)	693 (95.9%)		
II	52 (4.6%)	30 (4.1%)	0.199	0.656
Dominant				
DD	710 (62.6%)	440 (60.9%)		
II + ID	424 (37.4%)	283 (39.1%)	0.575	0.448
Allele				
D	1792 (79.0%)	1133 (78.4%)		
I	476 (21.0%)	313 (21.6%)	0.229	0.632

Data are presented as number (%)

* Odds ratio (OR) = 1.644, 95% confidence interval (CI): 1.053–2.568

** OR = 1.280, 95% CI: 1.054–1.554

*** OR = 1.275, 95% CI: 1.082–1.502

The association between the combined genotypes of the C-1054T and 96-bp I/D polymorphisms and the risk of GDM was also estimated. Owing to the relatively small sample size of the 96-bp II and − 1054 TT homozygotes, we integrated these homozygotes into the heterozygous subgroups. The frequency of the II + ID/TT + CT combined genotype was higher in the GDM group than that in the control group (12.0 vs. 7.8%; *P* = 0.013, Table 3). The II + ID/TT + CT combined genotype was a risk factor for GDM when the wild-type combined genotype DD/CC was used as a reference in a multinomial logistic regression model, including age and pre-pregnancy BMI as covariates (OR = 1.676, 95% CI: 1.182–2.376, *P* = 0.004).

**Table 3:** Combined genotypes of *CYP*2E1 96-bp I/D and C-1054T variants in GDM and control women

Genotype combinations	Controls (n = 1134)	GDM (n = 723)	OR	95%CI	*P*
DD/CC	427 (37.7%)	249 (34.4%)	1.000	-	-
DD/TT + CT	283 (25.0%)	191 (26.4%)	1.182	0.919–1.519	0.192
II + ID/CC	335 (29.5%)	196 (27.1%)	1.052	0.824–1.345	0.682
II + ID/TT + CT	89 (7.8%)	87 (12.0%)	1.676	1.182–2.376	0.004

Data of genotype combinations are presented as number (%) of patients or controls

Chi-squared test: *x*^*2*^ = 10.695, *P* = 0.013. Odds ratio (OR) and 95% confidence interval (CI) were calculated in a multinomial logistic regression model including age and pre-pregnancy BMI as covariates, the DD/CC combined genotypes (wild-type) as the reference category

### **Effects of*CYP2E1*C-1054T and 96-bp I/D variation on clinical, metabolic, and oxidative stress indices**

As shown in Table 4, GDM patients carrying the TT + CT genotype had higher fasting Ins levels, HOMA-IR, and gestational age (*P* < 0.05), but lower TG and TG/HDL-C ratio (*P* < 0.05) than those with the CC genotype. No significant differences in oxidative stress indices were observed between the TT + CT and CC genotype subgroups in patients with GDM and controls (*P* > 0.05). In all subjects, the TT + CT genotype subgroup had higher fasting Ins levels and HOMA-IR (*P* < 0.05), but lower SBP (*P* = 0.025) than the CC genotype subgroup; GDM status was associated with most of the parameters (*P* < 0.05) and an obvious interaction between the C-1054T variant and GDM status was observed in these parameters (*P* < 0.05) except for SBP, DBP, TC, HDL-C, apoB, and TAC (*P* > 0.05).

**Table 4:** Clinical and biochemical parameters according to *CYP2E1* C-1054T genotypes in GDM and control women

	Controls				GDM			All subjects			
	CC (n = 762)		TT + CT (n = 40 + 332)		CC (n = 445)	TT + CT (n = 41 + 237)		CC (n = 1207)	TT + CT (n = 81 + 569)	*P* ^*1*^	*P* ^*2*^
**Clinical characteristics**											
Age (years)	35.54 ± 3.76		35.52 ± 3.51		35.41 ± 4.04	35.92 ± 4.00		35.49 ± 3.87	35.69 ± 3.73		
Pre-pregnancy BMI (kg/m2) (kg/m2)	21.14 ± 2.71		21.47 ± 2.62		22.18 ± 2.93	22.42 ± 2.94		21.53 ± 2.83	21.89 ± 2.81		
Delivery BMI (kg/m2)	26.62 ± 2.73		26.95 ± 2.66		26.68 ± 2.91	27.08 ± 3.55		26.64 ± 2.80	27.01 ± 3.08		
Weight gain during pregnancy (kg)	14.01 ± 4.45		13.92 ± 3.86		11.39 ± 4.10	11.67 ± 4.36		13.03 ± 4.50	12.93 ± 4.23	< 0.001	< 0.001
SBP (mmHg)	115.47 ± 10.15		114.69 ± 10.17		116.12 ± 11.28	115.02 ± 12.71		115.71 ± 10.58	114.83 ± 11.32^b^	0.912	0.160
DBP (mmHg)	72.35 ± 7.76		71.87 ± 8.47		73.02 ± 9.05	72.25 ± 8.96		72.60 ± 8.26	72.03 ± 8.68	0.311	0.267
Gestational age (days)	274.69 ± 6.53		274.94 ± 5.59		271.62 ± 15.04	273.18 ± 7.10^a^		273.56 ± 10.60	274.19 ± 6.34	< 0.001	< 0.001
Parity	1.63 ± 0.55		1.61 ± 0.54		1.56 ± 0.53	1.60 ± 0.53		1.60 ± 0.54	1.60 ± 0.53	0.009	0.049
Neonatal birth height (cm)	49.94 ± 2.00		49.72 ± 1.74		49.57 ± 1.84	49.68 ± 1.84		49.80 ± 1.95	49.70 ± 1.72	0.004	0.011
Neonatal birth weight (g)	3384.85 ± 381.70		3379.41 ± 364.91		3319.82 ± 449.29	3359.49 ± 430.78		3360.87 ± 408.95	3370.88 ± 394.26	0.001	0.005
Metabolic profile*											
Fasting Glu (mmol/L)	4.36 ± 0.44		4.34 ± 0.40		4.58 ± 0.69	4.68 ± 0.97		4.45 ± 0.56	4.49 ± 0.73	< 0.001	< 0.001
Fasting Ins (pmol/L)	71.39 ± 36.18		74.80 ± 35.49		97.02 ± 91.81	122.09 ± 210.78^a^		81.33 ± 64.94	95.77 ± 144.53^b^	< 0.001	< 0.001
HOMA-IR	2.02 ± 1.12		2.09 ± 1.04		3.04 ± 3.74	4.64 ± 13.85^a^		2.42 ± 2.54	3.22 ± 9.32^b^	< 0.001	< 0.001
TG (mmol/L)	3.61 ± 1.41		3.68 ± 1.40		4.01 ± 1.78	3.74 ± 1.50^a^		3.76 ± 1.57	3.71 ± 1.44	0.005	0.004
TC (mmol/L)	6.08 ± 1.09		6.01 ± 1.05		5.98 ± 1.09	5.93 ± 1.10		6.05 ± 1.09	5.97 ± 1.07	0.352	0.578
HDL-C (mmol/L)	1.99 ± 0.42		1.98 ± 0.39		1.95 ± 0.42	1.99 ± 0.45		1.98 ± 0.42	1.98 ± 0.42	0.997	0.834
LDL-C (mmol/L)	3.20 ± 0.94		3.13 ± 1.08		2.96 ± 0.88	2.98 ± 1.09		3.11 ± 0.93	3.07 ± 1.08	0.001	0.008
TG/HDL-C	1.91 ± 0.89		1.93 ± 0.85		2.17 ± 1.21	1.99 ± 0.90^a^		2.01 ± 1.03	1.96 ± 0.87	0.006	0.004
ApoA1 (g/L)	2.37 ± 0.46		2.38 ± 0.40		2.28 ± 0.40	2.32 ± 0.45		2.34 ± 0.44	2.36 ± 0.42	0.001	0.011
ApoB (g/L)	1.16 ± 0.27		1.13 ± 0.25		1.16 ± 0.26	1.15 ± 0.25		1.16 ± 0.26	1.14 ± 0.25	0.339	0.510
ApoB/apoA1	0.51 ± 0.15		0.49 ± 0.14		0.52 ± 0.13	0.51 ± 0.13		0.51 ± 0.14	0.50 ± 0.14	0.012	0.038
Oxidative stress parameters**											
TOS (µmol H_2_O_2_ Equiv./L)	20.91 ± 6.86		21.10 ± 7.21		26.25 ± 10.47	25.35 ± 10.72		23.14 ± 8.94	23.07 ± 9.25	< 0.001	< 0.001
TAC (mmol Trolox Equiv./L)	1.10 ± 0.19		1.12 ± 0.20		1.13 ± 0.21	1.11 ± 0.20		1.11 ± 0.20	1.11 ± 0.20	0.878	0.607
OSI	19.52 ± 7.22		19.05 ± 7.43		23.68 ± 10.76	22.72 ± 8.93		21.27 ± 9.11	20.75 ± 8.35	< 0.001	< 0.001
MDA (nmol/ml)	5.37 ± 1.21		5.36 ± 1.22		5.95 ± 1.51	5.76 ± 1.26		5.59 ± 1.37	5.54 ± 1.25	< 0.001	< 0.001

Values are presented as mean ± SD.

BMI, body mass index; SBP, systolic blood pressure; DBP, diastolic blood pressure; Glu, glucose; Ins, insulin; HOMA-IR, homeostatic model assessment of insulin resistance; TG, triglycerides; TC, total cholesterol; HDL-C, high-density lipoprotein cholesterol; LDL-C, low-density lipoprotein cholesterol; apo, apolipoprotein; TOS, total oxidant status; TAC, total antioxidant capacity; MDA, malondialdehyde; OSI, oxidative stress index

For the control and GDM groups, comparisons of all parameters were corrected for differences in age and pre-pregnancy BMI except the parameters of age and BMI.

For all subjects, a two-way univariate general linear model introducing both the genotypes and GDM status as independent variables, with age and pre-pregnancy BMI as covariates was performed. *P*: the effect of genotype; *P*^*1*^: the effect of GDM status; *P*^*2*^: the interaction of genotype and GDM status

^a^*P* < 0.05, compared with the *CC* genotype subgroup in the GDM group; ^b^*P* < 0.05, compared with the *CC* genotype subgroup in all subjects

*Controls (CC = 715, TT + CT = 39 + 317); GDM (CC = 415, TT + CT = 37 + 222); all subjects (CC = 1130, TT + CT = 76 + 539)

**Controls (CC = 563, TT + CT = 33 + 253); GDM (CC = 344, TT + CT = 29 + 184); all subjects (CC = 907, TT + CT = 62 + 437)

Regarding the 96-bp I/D polymorphisms (Table 5), participants in the control group with genotype II + ID had higher DBP and parity (*P* < 0.05) than those with the DD genotype. There were no significant differences in oxidative stress and metabolic indices between the II + ID and DD genotype subgroups in the GDM and control groups and all subjects (*P* > 0.05). However, similar to the C-1054T variant, GDM status and its interaction with the 96-bp I/D polymorphism were significantly associated with most of the parameters (*P* < 0.05) except for SBP, DBP, TC, HDL-C, apoB, and TAC (*P* > 0.05).

**Table 5:** Clinical and biochemical parameters according to *CYP2E1* 96-bp I/D genotypes in GDM and control women

	Controls					GDM			All subjects			
	DD (n = 710)		II + ID (n = 52 + 372)			DD (n = 440)	II + ID (n = 30 + 253)		DD (n = 1150)	II + ID (n = 82 + 625)	*P* ^*1*^	*P* ^*2*^
**Clinical characteristics**												
Age (years)	35.45 ± 3.78		35.68 ± 3.50			35.55 ± 4.00	35.69 ± 4.07		35.49 ± 3.86	35.68 ± 3.74		
Pre-pregnancy BMI (kg/m2) (kg/m2)	21.26 ± 2.78		21.23 ± 2.52			22.38 ± 2.90	22.10 ± 2.97		21.70 ± 2.88	21.59 ± 2.75		
Delivery BMI (kg/m2)	26.74 ± 2.75		26.71 ± 2.65			26.99 ± 3.29	26.60 ± 2.99		26.83 ± 2.97	26.67 ± 2.79		
Weight gain during pregnancy (kg)	13.89 ± 3.88		14.11 ± 4.83			11.58 ± 4.07	11.37 ± 4.40		12.99 ± 4.11	13.00 ± 4.85	< 0.001	< 0.001
SBP (mmHg)	115.09 ± 10.12		115.41 ± 10.22			115.84 ± 11.48	115.47 ± 12.44		115.38 ± 10.66	115.43 ± 11.15	0.972	0.879
DBP (mmHg)	71.79 ± 8.24		72.88 ± 7.54^a^			72.86 ± 8.52	72.52 ± 9.75		72.19 ± 8.36	72.73 ± 8.49	0.382	0.102
Gestational age (days)	274.75 ± 6.20		274.81 ± 6.30			272.73 ± 7.25	271.43 ± 17.99		273.98 ± 6.69	273.46 ± 12.49	< 0.001	< 0.001
Parity	1.56 ± 0.53		1.73 ± 0.55^a^			1.58 ± 0.53	1.58 ± 0.53		1.56 ± 0.53	1.66 ± 0.54	0.007	0.002
Neonatal birth height (cm)	49.84 ± 1.99		49.92 ± 1.78			49.64 ± 1.79	49.56 ± 1.92		49.76 ± 1.92	49.77 ± 1.85	0.003	0.024
Neonatal birth weight (g)	3379.48 ± 380.72		3389.08 ± 368.60			3346.69 ± 427.49	3317.14 ± 464.74		3366.93 ± 399.40	3360.24 ± 411.05	0.001	0.008
Metabolic profile*												
Fasting Glu (mmol/L)	4.36 ± 0.42		4.35 ± 0.44			4.62 ± 0.72	4.63 ± 0.94		4.46 ± 0.57	4.46 ± 0.70	< 0.001	< 0.001
Fasting Ins (pmol/L)	72.64 ± 36.46		72.37 ± 35.21			104.45 ± 139.18	110.08 ± 165.01		85.42 ± 93.97	88.06 ± 111.05	< 0.001	< 0.001
HOMA-IR	2.05 ± 1.10		2.05 ± 1.09			3.46 ± 6.36	3.97 ± 12.30		2.61 ± 4.18	2.84 ± 8.01	< 0.001	< 0.001
TG (mmol/L)	3.63 ± 1.42		3.64 ± 1.38			3.86 ± 1.64	3.98 ± 1.75		3.72 ± 1.51	3.77 ± 1.55	0.007	0.025
TC (mmol/L)	6.05 ± 1.07		6.07 ± 1.09			5.93 ± 1.07	6.01 ± 1.14		6.00 ± 1.07	6.05 ± 1.11	0.315	0.597
HDL-C (mmol/L)	1.98 ± 0.42		1.99 ± 0.39			1.97 ± 0.43	1.97 ± 0.44		1.98 ± 0.43	1.98 ± 0.41	0.974	0.972
LDL-C (mmol/L)	3.15 ± 0.93		3.22 ± 1.08			2.97 ± 1.01	2.97 ± 0.89		3.08 ± 0.96	3.12 ± 1.02	0.001	0.004
TG/HDL-C	1.92 ± 0.86		1.92 ± 0.90			2.07 ± 1.03	2.15 ± 1.21		1.98 ± 0.93	2.01 ± 1.04	0.008	0.019
ApoA1 (g/L)	2.37 ± 0.45		2.38 ± 0.41			2.29 ± 0.44	2.30 ± 0.38		2.34 ± 0.45	2.35 ± 0.40	0.001	0.016
ApoB (g/L)	1.15 ± 0.26		1.15 ± 0.26			1.15 ± 0.26	1.16 ± 0.25		1.15 ± 0.26	1.16 ± 0.26	0.378	0.695
ApoB/apoA1	0.50 ± 0.14		0.50 ± 0.15			0.51 ± 0.14	0.52 ± 0.13		0.51 ± 0.14	0.51 ± 0.14	0.016	0.110
Oxidative stress parameters**												
TOS (µmol H_2_O_2_ Equiv./L)	20.94 ± 7.01		21.05 ± 6.91			25.61 ± 10.30	26.43 ± 11.01		22.93 ± 8.87	23.45 ± 9.36	< 0.001	< 0.001
TAC (mmol Trolox Equiv./L)	1.10 ± 0.19		1.11 ± 0.19			1.11 ± 0.20	1.13 ± 0.22		1.11 ± 0.20	1.12 ± 0.20	0.897	0.741
OSI	19.34 ± 7.45		19.40 ± 6.99			22.87 ± 9.90	24.08 ± 10.44		20.84 ± 8.74	21.53 ± 9.02	< 0.001	< 0.001
MDA (nmol/ml)	5.37 ± 1.19		5.36 ± 1.26			5.87 ± 1.47	5.89 ± 1.34		5.57 ± 1.33	5.58 ± 1.32	< 0.001	< 0.001

Values are presented as mean ± SD.

BMI, body mass index; SBP, systolic blood pressure; DBP, diastolic blood pressure; Glu, glucose; Ins, insulin; HOMA-IR, homeostatic model assessment of insulin resistance; TG, triglycerides; TC, total cholesterol; HDL-C, high-density lipoprotein cholesterol; LDL-C, low-density lipoprotein cholesterol; apo, apolipoprotein; TOS, total oxidant status; TAC, total antioxidant capacity; MDA, malondialdehyde; OSI, oxidative stress index

For the control and GDM groups, comparisons of all parameters were corrected for differences in age and pre-pregnancy BMI except the parameters of age and BMI.

For all subjects, a two-way univariate general linear model introducing both the genotypes and GDM status as independent variables, with age and pre-pregnancy BMI as covariates was performed. *P*: the effect of genotype; *P*^*1*^: the effect of GDM status; *P*^*2*^: the interaction of genotype and GDM status

^a^*P* < 0.05, compared with the *CC* genotype subgroup in the control group

*Controls (DD = 674, II + ID = 48 + 349); GDM (DD = 414, II + ID = 29 + 231); all subjects (DD = 1088, II + ID = 77 + 580)

**Controls (DD = 540, II + ID = 35 + 274); GDM (DD = 356, II + ID = 24 + 177); all subjects (DD = 896, II + ID = 59 + 451)

Effects of the combined genotypes of *CYP2E1* 96-bp I/D and C-1054T polymorphisms on clinical and biochemical indices were shown in Table 6. In patients with GDM, compared with the DD/CC genotype subgroup, the DD/TT + CT genotype subgroup had higher fasting Glu levels (*P* = 0.046), while the II + ID/TT + CT genotype subgroup had higher fasting Ins, HOMA-IR, and gestational age (*P* < 0.05). Patients with the II + ID/CC genotype had higher TG and TG/HDL-C ratio than those with the DD/TT + CT or the II + ID/TT + CT genotype (*P* < 0.05), and higher DBP than those with the II + ID/TT + CT genotype (*P* = 0.042). In all subjects, the II + ID/TT + CT genotype subgroup had higher fasting Ins levels and HOMA-IR than the DD/CC genotype subgroup (*P* < 0.05), and higher BMI at delivery, gestational age, and HOMA-IR, but lower TG levels than the II + ID/CC genotype subgroup (*P* < 0.05); the DD/TT + CT genotype subgroup also had higher BMI at delivery but lower SBP and DBP (*P* < 0.05) than the II + ID/CC genotype subgroup (*P* < 0.05); there was an obvious interaction between the combined genotype variants and GDM status (*P* < 0.05) for weight gain during pregnancy, gestational age, parity, fasting Glu and Ins, HOMA-IR, TG, LDL-C, TG/HDL-C ratio, TOS, OSI, and MDA (*P* < 0.05).

**Table 6:** Clinical and biochemical parameters according to the combined genotypes of *CYP2E1* C-1054T and 96-bp I/D variants in GDM women and all subjects

	GDM					All subjects					
	DD/CC (n = 249)	DD/TT + CT (n = 191)	II + ID/CC (n = 196)	II + ID/TT + CT (n = 87)		DD/CC (n = 676)	DD/TT + CT (n = 474)	II + ID/CC (n = 531)	II + ID/TT + CT (n = 176)	*P* ^*1*^	*P* ^*2*^
**Clinical characteristics**											
Age (years)	35.25 ± 3.83	35.94 ± 4.19	35.61 ± 4.28	35.88 ± 3.58		35.42 ± 3.86	35.58 ± 3.87	35.58 ± 3.87	35.99 ± 3.32		
Pre-pregnancy BMI (kg/m2) (kg/m2)	22.36 ± 3.02	22.41 ± 2.75	21.95 ± 2.80	22.42 ± 3.33		21.62 ± 3.01	21.82 ± 2.68	21.43 ± 2.60	22.08 ± 3.11		
Delivery BMI (kg/m2)	26.90 ± 3.00	27.11 ± 3.63	26.41 ± 2.78	27.03 ± 3.39		26.75 ± 2.91	26.96 ± 3.05	26.51 ± 2.64^e^	27.15 ± 3.15^f^		
Weight gain during pregnancy (kg)	11.48 ± 3.94	11.72 ± 4.23	11.28 ± 4.30	11.56 ± 4.65		13.02 ± 4.08	12.95 ± 4.15	13.04 ± 4.97	12.86 ± 4.46	< 0.001	< 0.001
SBP (mmHg)	116.34 ± 10.07	115.19 ± 13.03	115.84 ± 12.67	114.64 ± 11.93		115.69 ± 10.00	114.93 ± 11.52	115.72 ± 11.27^e^	114.56 ± 10.79	0.918	0.584
DBP (mmHg)	72.98 ± 7.29	72.69 ± 9.17	73.08 ± 10.24	71.26 ± 8.45^c^		72.35 ± 7.96	71.98 ± 8.90	72.92 ± 8.63^e^	72.18 ± 8.07	0.328	0.104
Gestational age (days)	272.78 ± 7.05	272.68 ± 7.51	270.16 ± 21.16	274.28 ± 6.00^a, b, c^		273.92 ± 6.78	274.06 ± 6.57	273.10 ± 14.04	274.53 ± 5.67^d, f^	< 0.001	< 0.001
Parity	1.55 ± 0.54	1.61 ± 0.53	1.58 ± 0.53	1.58 ± 0.54		1.56 ± 0.53	1.57 ± 0.53	1.65 ± 0.55	1.69 ± 0.53	0.007	0.035
Neonatal birth height (cm)	49.59 ± 1.67	49.71 ± 1.93	49.54 ± 2.04	49.62 ± 1.63		49.78 ± 2.03	49.74 ± 1.74	49.83 ± 1.83	49.61 ± 1.88	0.004	0.093
Neonatal birth weight (g)	3327.71 ± 411.28	3371.37 ± 447.58	3309.77 ± 494.39	3333.56 ± 392.80		3361.64 ± 4.4.69	3374.44 ± 392.07	3359.89 ± 414.70	3361.29 ± 401.07	0.001	0.061
Metabolic profile*											
Fasting Glu (mmol/L)	4.54 ± 0.63	4.72 ± 0.81^a^	4.64 ± 0.77	4.59 ± 1.26		4.44 ± 0.52	4.50 ± 0.64	4.46 ± 0.61	4.47 ± 0.94	< 0.001	< 0.001
Fasting Ins (pmol/L)	93.76 ± 88.20	118.20 ± 184.60	101.12 ± 96.33	131.34 ± 264.15^a^		80.01 ± 63.23	93.13 ± 124.61	83.09 ± 67.13	103.00 ± 189.61^d^	< 0.001	< 0.001
HOMA-IR	2.88 ± 3.61	4.21 ± 8.68	3.25 ± 3.91	5.66 ± 21.71 ^a,^		2.35 ± 2.46	2.98 ± 5.77	2.50 ± 2.64	3.87 ± 15.37 ^d, f^	< 0.001	< 0.001
TG (mmol/L)	3.92 ± 1.66	3.79 ± 1.61	4.13 ± 1.92^b^	3.62 ± 1.22^c^		3.71 ± 1.50	3.74 ± 1.52	3.82 ± 1.65	3.62 ± 1.20^f^	0.005	0.011
TC (mmol/L)	5.92 ± 1.04	5.94 ± 1.11	6.06 ± 1.11	5.89 ± 1.08		6.02 ± 1.06	5.99 ± 1.08	6.08 ± 1.13	5.94 ± 1.03	0.354	0.790
HDL-C (mmol/L)	1.95 ± 0.42	1.98 ± 0.44	1.96 ± 0.43	1.99 ± 0.47		1.97 ± 0.43	1.99 ± 0.41	1.98 ± 0.41	1.97 ± 0.42	0.985	0.977
LDL-C (mmol/L)	2.94 ± 0.87	3.01 ± 1.18	3.00 ± 0.90	2.92 ± 0.84		3.10 ± 0.91	3.05 ± 1.03	3.12 ± 0.94	3.11 ± 1.21	0.001	0.028
TG/HDL-C	2.12 ± 1.11	2.01 ± 0.91	2.23 ± 1.32 ^b^	1.96 ± 0.88 ^c^		1.98 ± 0.96	1.96 ± 0.89	2.04 ± 1.11	1.93 ± 0.81	0.006	0.017
ApoA1 (g/L)	2.27 ± 0.41	2.32 ± 0.47	2.30 ± 0.38	2.32 ± 0.40		2.33 ± 0.46	2.36 ± 0.43	2.35 ± 0.41	2.35 ± 0.39	0.001	0.122
ApoB (g/L)	1.14 ± 0.27	1.15 ± 0.26	1.18 ± 0.26	1.13 ± 0.23		1.15 ± 0.26	1.14 ± 0.26	1.16 ± 0.27	1.13 ± 0.23	0.338	0.751
ApoB/apoA1	0.51 ± 0.14	0.51 ± 0.14	0.52 ± 0.13	0.50 ± 0.12		0.51 ± 0.14	0.50 ± 0.14	0.51 ± 0.15	0.49 ± 0.12	0.012	0.230
Oxidative stress parameters**											
TOS (µmol H2O2 Equiv./L)	26.23 ± 10.82	24.79 ± 9.54	26.29 ± 10.00	26.79 ± 13.27		22.99 ± 9.13	22.85 ± 8.50	23.36 ± 8.67	23.74 ± 11.22	< 0.001	< 0.001
TAC (mmol Trolox Equiv./L)	1.13 ± 0.20	1.10 ± 0.20	1.13 ± 0.23	1.13 ± 0.18		1.10 ± 0.19	1.11 ± 0.20	1.12 ± 0.21	1.11 ± 0.19	0.889	0.366
OSI	23.24 ± 10.98	22.35 ± 8.25	24.25 ± 10.46	23.65 ± 10.46		21.08 ± 9.29	20.49 ± 7.91	21.53 ± 8.86	21.53 ± 9.53	< 0.001	< 0.001
MDA (nmol/ml)	5.92 ± 1.58	5.80 ± 1.32	5.99 ± 1.42	5.66 ± 1.10		5.58 ± 1.37	5.55 ± 1.28	5.61 ± 1.36	5.49 ± 1.16	< 0.001	< 0.001

Values are presented as mean ± SD.

BMI, body mass index; SBP, systolic blood pressure; DBP, diastolic blood pressure; Glu, glucose; Ins, insulin; HOMA-IR, homeostatic model assessment of insulin resistance; TG, triglycerides; TC, total cholesterol; HDL-C, high-density lipoprotein cholesterol; LDL-C, low-density lipoprotein cholesterol; apo, apolipoprotein; TOS, total oxidant status; TAC, total antioxidant capacity; MDA, malondialdehyde; OSI, oxidative stress index

For the control and GDM groups, comparisons of all parameters were corrected for differences in age and pre-pregnancy BMI except the parameters of age and BMI.

For all subjects, a two-way univariate general linear model introducing both the genotypes and GDM status as independent variables, with age and pre-pregnancy BMI as covariates was performed. *P*: the effect of genotype; *P*^*1*^: the effect of GDM status; *P*^*2*^: the interaction of genotype and GDM status

^a^*P* < 0.05, compared with the DD/CC genotype subgroup in the GDM group; ^b^*P* < 0.05, compared with the DD/TT + CT genotype subgroup in the GDM group; ^c^*P* < 0.05, compared with the II + ID/CC genotype subgroup in the GDM group; ^d^*P* < 0.05, compared with the DD/CC genotype subgroup in all subjects; ^e^*P* < 0.05, compared with the DD/TT + CT genotype subgroup in all subjects; ^f^*P* < 0.05, compared with the II + ID/CC genotype subgroup in all subjects

*GDM (DD/CC = 234, DD/TT + CT = 180, II + ID/CC = 181, II + ID/TT + CT = 79); all subjects (DD/CC = 639, DD/TT + CT = 449, II + ID/CC = 491, II + ID/TT + CT = 166)

**GDM (DD/CC = 201, DD/TT + CT = 155, II + ID/CC = 143, II + ID/TT + CT = 58); all subjects (DD/CC = 526, DD/TT + CT = 370, II + ID/CC = 381, II + ID/TT + CT = 129)

## Discussion

To the best of our knowledge, this study is the first to demonstrate that the C-1054T variant, but not the 96-bp I/D variant in *CYP2E1* is associated with GDM risk according to genotype, recessive, dominant, and allele models in the Chinese population. We also showed that the combined genotype II + ID/TT + CT further increased the risk of GDM when the combined genotype DD/CC was used as the reference. Moreover, we found that GDM patients carrying the T allele of C-1054T variation had lower TG levels and TG/HDL-C ratio but higher fasting insulin and HOMA-IR than those carrying the CC genotype; GDM patients carrying the DD/TT + CT or II + ID/TT + CT combined genotype had higher fasting Glu or Ins levels and HOMA-IR values, and those with the II + ID/CC genotype had higher TG and TG/HDL-C ratio, implying that the C-1054T and 96-bp I/D variants in *CYP2E1* may be linked to lipid metabolism, hyperinsulinemia, and insulin resistance in the patients.

The protein levels and activities of CYP2E1 are influenced by genetic variations, environmental factors, and disease status [23, 36]. Moreover, the distribution of *CYP2E1* genetic variations shows clear ethnic differences [23]. Therefore, investigating *CYP2E1* genetic variations in patients with GDM may help identify genetic predispositions and elucidate the etiopathogenesis of GDM.

Reports regarding the effect of C-1054T variation on the function and expression of CYP2E1 are inconsistent. The T allele (RsaI c2) was reported to increase transcription of the *CYP2E1* gene [30] but was associated with low enzyme activity [40] and low inducible activity after ethanol induction [41] or did not influence enzyme activity [42]. The study found that the T allele frequency of the C-1054T variant ranges from 17.7 to 25.0% in the East Asian population [23, 32], and is higher than that in Caucasians (4.0%) and Iranians (1.5%) [23, 43]. The T allele is a genetic risk factor for colorectal cancer in the Brazilian population [31], hepatitis B-related hepatocellular carcinoma [44], and PCOS in Chinese women [27]. However, it is a protective factor for bladder cancer in Asian populations [45] and patients with lung cancer or drug-related liver damage [25, 35]. A study reported that the T allele is related to lower birth weight of newborns whose maternal disinfection by-products are exposed during gestation [34]. In this study, we demonstrated that the T allele of the C-1054T SNP is a genetic risk factor for GDM in the Chinese population. Moreover, we found that compared with GDM patients carrying the CC genotype, those carrying the T allele had lower TG levels and TG/HDL-C ratio but higher fasting insulin and HOMA-IR. This implies that the C-1054→T genetic variation may affect lipid metabolism and aggravate insulin resistance in patients. Nevertheless, the underlying mechanisms, including whether the T allele increases the risk of GDM by influencing xenobiotic degradation, should be further explored.

The I allele of the 96-bp I/D variation in *CYP2E1* enhances the transcriptional activity of *CYP2E1* [28]. Studies have found a relatively high frequency of the 96-bp I allele in Asians (15−23.7%) [29, 32], but it is relatively low in African-Americans (10%) and Caucasians (2%) [29]. Genotype II or allele I carriers are associated with a higher risk of drug-induced liver injury [35] and colorectal cancer [32]. In this study, the I allele frequency was 21.2% in all participants. No significant differences were observed between the GDM and control groups according to the different genetic models for the 96-bp I/D variation. However, we found that the II + ID/TT + CT combined genotype of the 96-bp I/D and C-1054T polymorphisms further increased the risk of GDM when the reference genotype DD/CC was used. We also demonstrated that GDM patients carrying the DD/TT + CT or II + ID/TT + CT combined genotype had higher fasting Glu, Ins, and HOMA-IR, and those with the II + ID/CC combined genotype had higher TG levels and TG/HDL-C ratio, suggesting that these two genetic variants may be involved in insulin resistance and dyslipidemia in the patients. Further research is required to elucidate this issue and its underlying mechanisms.

Placental dysfunction plays an important role in the pathogenesis of GDM [19–21, 46]. An increase in maternal pre-pregnancy BMI, glucose levels, and weight gain during pregnancy are associated with the abnormal expression of placental hormones and cytokines [19–21]. Upregulation of placental inflammatory cytokines, oxidative stress-related genetic variants (myeloperoxidase G-463 A, *CYBA* C242T, *CYP2E1* C-1054T, etc.), glycation and oxidation of proteins caused by hyperglycemia were associated with unfavourable metabolic profiles, insulin resistance, increased oxidative stress, and state of chronic inflammation in patients with GDM, which might increase the risk of adverse perinatal outcomes [6, 8, 11, 20, 21, 47]. In contrast with most of published data in literature [2, 6, 21], in the present study, we found that the gestational weight gain and the birth height and weight of neonates were lower, whereas the incidence of macrosomia were similar in the GDM group than in the control group. One explanation might be that the patients with GDM recruited in our study were subjected to standardized and good pregnancy health care, the blood glucose of most patients with GDM were controlled to an ideal level only by diet control and exercise, except for approximately 10% of patients who required insulin therapy. Our results support the findings that decreased gestational weight gain and continuous glucose monitoring use in pregnancy may help to prevent the occurrence of GDM and improve the treatment and outcomes of GDM [1, 7, 48].

This study has some limitations. First, because of the comparatively low frequencies of minor allelic homozygosity (96-bp II and − 1054 TT), we could not analyze them in the subgroup analysis. A larger sample size is required to evaluate the dose-dependent genotype characteristics. Second, we did not determine the levels or activities of CYP2E1. It may be helpful to further analyze enzyme function to reveal the association between genetic variation and GDM risk. Third, based on the function of CYP2E1, further analysis of the state of xenobiotics in the GDM and control groups may help determine the potential mechanism underlying *CYP2E1* genotypic variations and risk of GDM. Fourth, we did not measure metabolic or oxidative parameters in some subjects due to inadequate sample volume or samples with bilirubin or hemolysis, which might influence the power of these parameters or result in the absence of statistical significance.

## Conclusions

This study demonstrated that the *CYP2E1* genetic polymorphism C-1054T, but not 96-bp I/D, is associated with an increased risk of GDM in the Chinese population. We also showed that the combined genotype II + ID/TT + CT of these two polymorphisms was associated with a higher risk of GDM. Furthermore, we found that GDM patients with the T allele of the C-1054T variant had more serious insulin resistance. Our findings provide new evidence that genetic variants of xenobiotic metabolism-related enzymes may contribute to the pathogenesis of GDM.

## Data Availability

The datasets generated and/or analyzed during the current study are available from the corresponding author upon reasonable request.

## Abbreviations

GDM: gestational diabetes mellitus
CYP2E1: cytochrome P450 2E1
BMI: body mass index
SBP: systolic blood pressure
DBP: diastolic blood pressure
Glu: glucose
Ins: insulin
HOMA-IR: homeostatic model assessment of insulin resistance
TG: triglycerides
TC: total cholesterol
HDL-C: high-density lipoprotein cholesterol
LDL-C: low-density lipoprotein cholesterol
apo: apolipoprotein
TOS: total oxidant status
TAC: total antioxidant capacity
MDA: malondialdehyde
OSI: oxidative stress index
OR: Odds ratio
CI: confidence interval
